# Ejaculation Sparing Thulium Laser Enucleation of the Prostate: An Observational Prospective Study

**DOI:** 10.3390/jcm11216365

**Published:** 2022-10-28

**Authors:** Francesco Trama, Giovanni Di Lauro, Ester Illiano, Fabrizio Iacono, Leo Romis, Salvatore Mordente, Maria Rosaria Nugnes, Stefano Lai, Felice Crocetto, Biagio Barone, Francesco Paolo Calace, Giuseppe Romeo, Elisabetta Costantini

**Affiliations:** 1 Andrology and Urogynecology Clinic, Santa Maria Terni Hospital, University of Perugia, 06123 Perugia, Italy; 2 Urology Complex Unit–ASL Napoli 2 Nord ‘Santa Maria delle Grazie’ Hospital, 80078 Pozzuoli, Italy; 3 Department of General and Specialized Surgeries, Renal Transplantation, Nephrology, Intensive Care and Pain Management, University of Federico II, 80138 Naples, Italy; 4 Urology Unit, Department of Woman, Child and General and Specialized Surgery, University of Campania “Luigi Vanvitelli”, 81100 Naples, Italy; 5 Urology Department, Azienda Ospedaliera di Rilievo Nazionale Antonio Cardarelli, 80131 Naples, Italy

**Keywords:** lower urinary tract symptoms, thulium, benign prostatic hyperplasia, ejaculation-sparing technique, sexual function

## Abstract

Benign prostatic hypertrophy (BPH) is a condition that appears with advancing age and affects 1/3 of men over 50 years, resulting in filling and emptying symptoms. One of the main limitations of endoscopic techniques for BPH is the occurrence of retrograde ejaculation. The purpose of this prospective observational study is to evaluate the efficacy and feasibility of ejaculation-sparing thulium laser enucleation of the prostate (ES-ThuLEP) in the treatment of BPH-related LUTS and the preservation of ejaculation. Sexually active patients with BPH were enrolled and followed up with at 3, 6, and 12 months after surgery. Personal and pharmacological histories were collected, while three standardized questionnaires—the International Index of Erectile Function short form (IIEF-5), the International Consultation on Incontinence Questionnaire for Male Sexual Matters Associated with Lower Urinary Tract Symptoms Module (ICIQ—MLUTSsex), and the International Prostatic Symptom Score (IPSS)—were administered. In addition, all patients underwent uroflowmetry and an assessment of post-void residual volume (PVR). A total of 53 patients were enrolled. A statistically significant improvement in the IPSS score, maximum flow (Qmax), and post-void volume (PVR) at 3 months, 6 months, and 12 months after surgery was found (*p* < 0.05), while no statistically significant differences were reported between IIEF-5 scores before and after surgery. A total of 48 patients (88.6%) had preserved ejaculation at 3 months, while 92.4% and 94.3% of patients reported preserved ejaculation at 6 and 12 months, respectively. Nevertheless, some degree of hypoposia was referred, at 3, 6, and 12 months, by 43.7%, 30.6%, and 13.2% of patients, respectively. The ES-ThuLEP technique successfully preserved ejaculation in over 90% of patients, representing an ejaculation-sparing alternative in the treatment of BPH.

## 1. Introduction

Benign prostatic hypertrophy (BPH) is a medical condition characterized by the enlargement of the prostate, particularly frequent in men >50 years, and caused by the prevalent production of prostatic growth-stimulating factors over inhibiting factors [1]. In the early stages, the condition is characterized by the formation of nodules at the periurethral site, consisting of stromal and parenchymal elements that successively increase in number and size, deforming the urethra and obstructing the outflow of urine [2]. As result, filling and emptying symptoms could be present. In 15% of cases, the severity of the resulting obstructive uropathy symptoms requires surgery [3]. In recent years, the surgical treatment of BPH has been enriched by a multitude of endoscopic, laparoscopic, and robotic surgical techniques. Nowadays, transurethral resection of the prostate (TURP) remains the gold standard treatment, even though it is associated with non-negligible morbidity—estimated as 2.3–22% of the overall complication rate—and a loss-of-ejaculation rate of 78% [4,5]. A variety of lasers have helped to reduce patient hospitalization, blood loss, and catheterization time. This is due to the natural thermal effect of lasers, which permits surgeons to achieve point coagulation and hemostasis [6].

According to the EAU guidelines, thulium laser enucleation of the prostate (ThuLEP) is an endoscopic surgical technique that reported comparable results to TURP in the treatment of BPH while yielding less blood loss [7]. Thulium (Tm:YAG) was introduced in the urological landscape in 2005. Its unique characteristics are the 2013 nm wavelength and the continuous delivery of energy that allows minimal tissue penetration and point coagulation, resulting in excellent hemostasis [8]. For these reasons, thulium lasers have enormous potential in prostatic surgery. Nevertheless, as with all the other endoscopic and surgical BPH treatments (including open, laparoscopic, and robotic-assisted surgeries), the ThuLEP is similarly bounded by the loss of ejaculation after surgery [9]. The only recommended ejaculation-sparing procedure, currently, is a device—called UROLIFT—which permits the compression of the lateral prostatic lobes without removing the hyperplastic tissue. Although it could represent a valid alternative, especially in frail and elderly patients, the procedure is not recommended for prostates over 80 g or with a third lobe, and it presents, overall, worse functional outcomes compared to TURP [10,11]. Several pieces of evidence have demonstrated the importance of the prostatic tissue surrounding the veru montanum for anterograde ejaculation, with a pivotal role played by the musculus ejaculatorious, a thin longitudinal strain of muscle fibers extending from the ejaculatory ducts to the urethral sphincter [12]. Due to these premises, supramontanal and paracollicular tissue-sparing techniques have been developed in order to maintain anterograde ejaculation in patients undergoing TURP and photo-selective vaporization of the prostate [13,14]. More recently, ejaculation-sparing techniques have also been utilized, through the use of thulium lasers, thus overcoming size limitations and providing comparable improvement in relieving BPH symptoms [15,16]. According to the reported findings, the purpose of this work was to evaluate the sexual and functional outcomes of ejaculation-sparing ThuLEP (ES-ThuLEP) in patients with BPH wishing to maintain ejaculatory function. 

## 2. Materials and Methods

The study was designed as a single-center observational study, performed from December 2020 to March 2021. All procedures involving human participants were in accordance with the ethical standards of the institution and with the 1964 Declaration of Helsinki and its later amendments or comparable ethical standards. The study was approved by the local ethics committee (Perugia University 2910/22). Sexually active patients who suffered from filling and voiding LUTS secondary to BPH were included. Exclusion criteria were: age >80 years; International Index of Erectile Function-5 (IIEF-5) score <16; previous endoscopic or open pelvic surgery for benign prostatic hypertrophy; psychiatric disorders or urological-oncological problems; not sexually active patients; previous ejaculation-sparing surgery; suspected prostate cancer at digital rectal exploration (DRE) or elevated total PSA levels; alpha-blocker therapy in the previous three months or not discontinued for at least 21 days before the time of enrollment. All subjects underwent, at the time of enrollment, a thorough urological examination, which included DRE, serum PSA assay, uroflowmetry with the evaluation of post-micturition residual (PVR), and transrectal ultrasound of the prostate for the calculation of prostate volumetry. Additionally, the IPSS questionnaire for the evaluation of LUTS, the IIEF-5 questionnaire for the evaluation of erection quality, and the International Consultation of Incontinence Questionnaire (ICIQ)—MLUTSsex (Question 3a “Do you have ejaculation of seminal fluid?” and question 4a “Do you experience pain or discomfort during ejaculation?”) were administered [17,18,19]. All questionnaires were validated in the Italian language. All eligible patients underwent ES-ThuLEP surgery. Patients underwent urological follow-up visits at 3, 6, and 12 months, during which the questionnaires were re-administered. 

### 2.1. Statistical Analysis

Statistical analysis was performed using the McNamar chi-square test to compare paired categorical variables, while paired *t*-tests were used for continuous parametric variables. The Wilcoxon and Kruskal–Wallis tests were used for the mean and median values, respectively, of quantitative variables. All calculations were performed using IBM-SPSS^®^ version 22.0 (IBM Corp., Armonk, NY, USA, 2013). A *p*-value < 0.05 was considered to be statistically significant.

### 2.2. Surgical Technique

The first step consisted of an Omega-like incision of 1 cm, cranially to the verumontanum. Successively, the incision was extended to the two lateral lobes, taking care to preserve a small amount of tissue from the lateral lobes at the level of the prostatic apex near the verumontanum, in order to preserve the integrity of the ejaculatory muscles and the positioning of the verumontanum below the sphincter, which allow for the passage of semen into the bulbar urethra. The next steps were the exposure of the surgical capsule and the enucleation of the left and right lobes and the central adenoma. The enucleated tissue was finally morcellated. All surgical procedures were performed by a single operator, with at least five years of experience in BPH thulium laser surgery treatment (>500 procedures performed), and utilizing the RevoLix Thulium:YAG (LISA Laser products, Berlin, Germany) with a 2-micron continuous wave. The laser fiber used was a multiple-use optical 550 micron (RigiFib, LISA laser Products), while the energy levels used for the enucleation and coagulation were 60 W and 48 W, respectively. The laser fiber was used in a Karl–Stortz 26 French continuous-flow resectoscope, with irrigation using a 0.9% sodium chloride solution. 

Morcellation was performed with the Piranha morcellator at 450 rpm (Richard Wolf, Knittlingen, Germany).

## 3. Results

**Figure 1:**
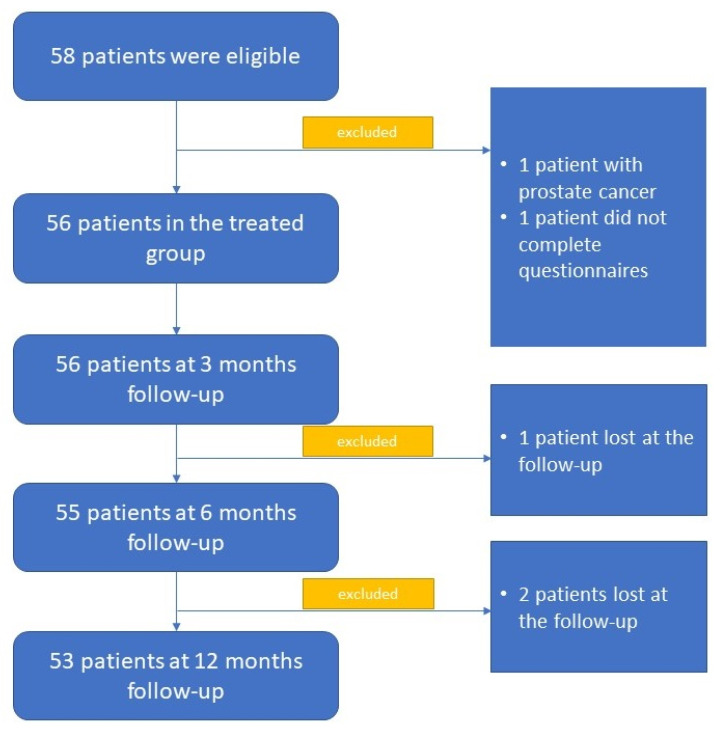
Flowchart reporting patients selected for participation and followed up with.

Fifty-eight subjects were declared eligible. Of these, three patients refused to continue the required follow-ups, one subject was diagnosed with prostate cancer, and finally, one subject did not complete all the required questionnaires (Figure 1).

In the end, 53 subjects, with a mean age of 60 ± 11.5 years, were enrolled. Table 1 shows the preoperative characteristics of the patients.

The intra-operative characteristics and postoperative complications of the patients involved are shown in Table 2 and Table 3. None of the patients underwent re-intervention during the follow-up period.

Table 4 shows the parameters measured during the follow-up. A statistically significant improvement was found for Qmax (*p* < 0.001), Qave (*p* < 0.001), ISR (*p* < 0.001), IPSS score (*p* < 0.001), and total PSA value (*p* < 0.001) up to 12 months after surgery. No statistically significant differences were demonstrated regarding the voided volume during uroflowmetric examination (*p* > 0.05) or in the IIEF-5 score (*p* > 0.05).

Regarding ejaculation, 48 patients (88.6%) reported preserved ejaculation at the 3-month follow-up; 92.4% at the 6-month follow-up, and 94.3% at the 12-month follow-up.

Of the 48 subjects reporting preserved ejaculation at 3 months, 43.7% reported that the amount was reduced; at 6 months, 30.6% of the subjects with preserved ejaculation reported a reduced amount of semen; and at 12 months, 7 subjects with preserved ejaculation (13.2%) reported a reduced amount of semen.

Regarding ejaculation, 48 patients (88.6%) had preserved ejaculation at 3 months after surgery, 51 patients (92.4%) reported preserved ejaculation at 6 months after surgery, and 52 patients (94.3%) reported preserved ejaculation at 12 months after surgery.

Among patients reporting preserved ejaculation at 3 months, 43.7% reported a reduced amount of semen; at 6 months, the percentage of patients decreased to 30.6% and, at 12 months, only 13.2% of patients with preserved ejaculation reported a decreased amount of semen. 

## 4. Discussion

Benign prostatic hypertrophy (BPH) is the main cause of LUTS [20]. Surgery is considered the most effective treatment, with TURP playing a prominent role in this area in recent years [20]. On the contrary, the use of laser technology in the treatment of BPH has developed, widely due to its vast potential and ease of use [21]. In fact, lasers reduce morbidity and shorten the catheterization period, with less blood loss and fewer days of hospitalization [22]. 

In particular, thulium allows for efficient vapoenucleation and, at the same time, effective hemostasis, with an absorption capacity of the surrounding tissues that is lower than other lasers used in the treatment of BPH, such as holmium [22].

Zhang et al., in 2012, compared the ThuLEP technique with the HoLEP technique, conducting a prospective randomized trial involving a total of 133 patients with a maximum prostate size of 80 g and a median follow-up time of 18 months. No significant differences regarding urinary symptoms, catheterization time, or the amount of enucleated prostate were found, while a lower rate of blood loss was reported for the thulium laser [23]. 

Pirola et al., in 2018, published a nonrandomized retrospective study in which ThuLEP and HoLEP were compared; they reported similar outcomes in terms of urinary symptomatology improvement, while reduced blood loss was found for ThuLEP [24]. Similarly, in 2020, Zhang et al. analyzed a sample of 116 subjects undergoing ThuLEP or HoLEP, for a maximum follow-up period of 18 months, and found that the thulium laser was superior to the holmium laser in terms of operation and enucleation time [25]. Finally, Bozzini et al., in 2020, published a prospective study involving 236 patients randomized to either the ThuLEP or HoLEP procedure; they reported equal efficacy and safety, although the ThuLEP procedure was reported to have reduced risk of postoperative complications and blood loss [22]. Nevertheless, the limitation of all endoscopic resection and enucleation techniques for the treatment of BPH is the loss of ejaculation [26]. In fact, this is among the main reasons discouraging men from undergoing surgery—thus resulting in the onset of detrusor hypertrophy, bladder lithiasis, and up to, in some cases, bilateral hydroureteronephrosis— and impairing renal function [27]. 

Different minimally invasive surgical techniques have been developed that simultaneously allow for good cervico-urethral unclogging while maintaining ejaculation (Urolift, iTIND, Acquablation, and Rezum), but there are limited data regarding the application of these techniques, especially in patients with an obstructive third lobe [28]. In addition, there are no data regarding efficacy in large prostates. For these reasons, the EAU guidelines recommend long-term studies to verify their effectiveness for urinary symptoms and their side effects [7]. 

On the contrary, the use of an enucleation surgery that provides good efficacy in terms of urinary symptomatology, combined with a technique that preserves anterograde ejaculation, seems to result in better urinary symptomatology and sexual satisfaction. 

In 1998, Ronzoni et al. published the first pilot study involving the TURP ejaculation-sparing technique, recruiting 45 patients in which more than 1 cm of prostatic urethra above the verumontanum was spared. Approximately 80% of the treated patients successfully maintained anterograde ejaculation [29].

Kim et al. published a study involving 52 patients, half of whom underwent ES-HoLEP; in this case, only 46.2% of patients maintained ejaculation [30]. The conflicting results, published by Kim, with respect to our study, are perhaps attributable to the difference between thulium and holmium lasers. In fact, the thulium laser, characterized by minor tissue penetration and a continuous energy wave, could better preserve structures that are believed to be critical for maintaining ejaculation from irreversible damage. Conversely, the pulsed wave of holmium could necessitate greater traction for the dissection of the tissues, thus increasing the possibility of secondary mechanical damage to the ejaculatory tissues. A larger study by Talab et al. analyzed 160 patients who underwent the EP-PVP technique (sparing the supramuntanar and paracullicular tissues), for a median follow-up period of 74 months, in order to determine the performance of the EP-PVP technique. This study reported an ejaculation-sparing rate of 86.6% in concomitance with the improvement of urinary parameters [13]. Similar findings were reported by Brant et al., which reported, following the ejaculatory hood-sparing transurethral vaporization of the prostate, the preservation of anterograde ejaculation in 80% of patients [31]. These findings demonstrated the contiguity between the prostatic apex and the ejaculatory ducts. As result, by sparing this area from treatment, it is possible to spare the ejaculatory ducts and the surrounding muscle fibers in a manner that ensures the anatomy of the prostatic urethra during ejaculation is unaltered.

More recently, Bozzini et al., in a prospective study involving 283 patients undergoing ES-ThuLEP, obtained comforting results. In fact, 203 patients (71%) reported anterograde ejaculation at 3 months after surgery, which increased to 219 patients (77%) at 6 months after the procedure, while achieving statistically significant improvements in their Qmax and IPSS scores [15].

The limitations of this study are several: First is the absence of a control group, as the ES-ThuLEP was intended to be considered in a comparison with other endoscopic techniques, such as the standard TURP. The second limitation is the absence of hormonal assessments of the patients involved. Thirdly, the sample size, compared to the prevalence of BPH in the overall population, is small. The fourth limitation is in the nature of the questionnaires, which are intrinsically bounded by self-assessment limitations. In addition, it has to be reported that the small sample size did not permit the analysis of the preoperative and perioperative factors associated with preserved ejaculation, considering that the majority of the patients involved reported maintained anterograde ejaculation. Nevertheless, the main strengths of our study are the long follow-up period (12 months), the minimal loss of patients, and the utilization of a single surgeon, which minimized bias related to different surgeons and learning curves.

Prospective randomized trials are, however, needed to validate the results of this modified surgical technique.

## 5. Conclusions

The ES-ThuLEP is an endoscopic surgical technique that can be proposed for patients with LUTS caused by BPH who have good erectile function for the improvement of urinary functional outcomes while preserving ejaculation.

## Figures and Tables

**Table 1:** Preoperative clinical characteristics. SD, standard deviation; PVR, post-voided residue; IPSS, international prostatic symptom score; IIEF, International Index of Erectile Function.

Age, years (mean ± SD)	60 ± 11.5
PSA, ng/mL (mean ± SD)	3.9 ± 2.3
Prostate volume, cc (mean ± SD)	88.8 ± 32.2
Previous alpha-blocker therapy, *n* (%)	64.2%
Previous alpha blocker + 5 alpha-reductase inhibitor, *n* (%)	24.5%
Bladder catheter, *n* (%)	5.6%
Qmax, mL/s (mean ± SD)	7.7 ± 2.5
Qave, mL/s (mean ± SD)	5.2 ± 2
Voided volume, mL (mean ± SD)	294.8 ± 87.5
PVR, mL (mean ± SD)	82.1 ± 37.1
IPSS score (mean ± SD)	25.7 ± 4.2
IIEF-5 score (mean ± SD)	21.3 ± 2.9

**Table 2:** Intra- and peri-operative parameters.

**Intra-Operative Parameters**	
Operative time, minutes (mean ± SD)	147.7 ± 67.8
Hb drop, g/dL	0.9 ± 0.7
Enucleated tissue weight, g (mean ± SD)	31.2 ± 14.2
Use of thulium laser, minutes (mean ± SD)	72.2 ± 11.3
**Peri-Operative Parameters**	
Hospitalitazion, days (mean ± SD)	3.2 ± 1.1
Stop bladder irrigation, days (mean ± SD)	1.9 ± 0.9
Catheter removal, days (mean ± SD)	2.8 ± 0.6

**Table 3:** Post-operative complications encountered during follow-up.

Acute urinary retention, *n* (%)	2 (3.7%)
Bladder neck sclerosis, *n* (%)	1 (1.8%)
Haematospermia at 3 months, *n* (%)	5 (9.4%)
Haematospermia at 6 months, *n* (%)	3 (5.6%)
Haematospermia at 9 months, *n* (%)	0 (0%)
Haematospermia at 12 months, *n* (%)	0 (0%)
Prostate adenocarcinoma, *n* (%)	1 (1.8%)

**Table 4:** Pre- and post-operative results at 3, 6, and 12 months after surgery. mL, milliliter; IPSS, International Prostatic Symptom Score; IIEF, International Index of Erectile Function.

	Baseline	3 Months	6 Months	12 Months	*p*-Value
Qmax, mL/s (mean ± SD)	7.7 ± 2.5	20.8 ± 4.1	21.7 ± 4	20 ± 3.7	<0.001
Qave, mL/s (mean ± SD)	5.2 ± 2	12.6 ± 3.2	14.3 ± 3.7	13.8 ± 3.8	<0.001
PSA level, ng/mL (mean ± SD)	3.9 ± 2.3	2.2 ± 1.3	2.3 ± 1.3	1.8 ± 1.4	<0.001
PVR, mL (mean ± SD)	82.1 ± 37.1	18.8 ± 12.3	18.6 ± 12.4	23.4 ± 14.9	<0.001
Voided volume, mL (mean ± SD)	294.8 ± 87.5	294.5 ± 80.7	301.9 ± 90.2	302.9 ± 97.7	0.85
IPSS score	25.7 ± 4.2	3.4 ± 2	5.6 ± 3.2	5.1 ± 3.4	<0.001
IIEF-5 score	21.3 ± 2.9	22.9 ± 3.8	20.4 ± 2.4	21.2 ± 2.6	0.052

## Data Availability

Not applicable.
